# Angiomatoid giant cellular blue nevus of vaginal wall associated with pregnancy

**DOI:** 10.1186/1746-1596-6-32

**Published:** 2011-04-08

**Authors:** Mubarak M Al-Shraim

**Affiliations:** 1 Department of Pathology, College of Medicine, King Khalid University, Saudi Arabia

**Keywords:** Cellular blue nevus, vagina, pregnancy, melanocytes

## Abstract

**Background:**

Blue nevi that arise from the Müllerian tract are rare melanocytic lesions. Several histopathologic variants of cellular blue nevi have been described. The angiomatoid variant is characterized by a vascular component, and is considered to be a rare variant. Few studies have explored the influence of pregnancy on melanocytic lesions.

**Case:**

A 29-year-old woman was presented with a pigmented vaginal lesion that increased gradually during pregnancy. A full term gynecologic examination showed a tumor mass protruding into the vaginal canal. The mass was resected during cesarean-section under the clinical impression of vaginal hemangioma.

**Result:**

Gross examination revealed a cystic mass measuring 6.0 × 4.3 × 3.5 cm, which was filled with dark friable material. Histologically, the mass showed a subepithelial cellular proliferation of heavily pigmented dendritic melanocytes with prominent vascular stroma. Cytologic pleomorphism, junctional activity, atypical mitosis, and necrosis were not found. The proliferation was immunoreactive for HMB-45, S-100 and melan-A, and non-immunoreactive for CD34, smooth muscle actin, and AE1/AE3. The MIB-1 proliferative index was less than 1%. The patient had a postoperative course without complication.

**Conclusions:**

Angiomatoid giant cellular blue nevus arising from the vagina during pregnancy is extremely rare. The low proliferative index and absence of cytologic pleomorphism, or necrosis, supports a benign biological behavior. Clinical follow-up showed no evidence of recurrence at one year after the resection of the mass.

## Introduction

Blue nevi are rare benign pigmented lesions that are derived from dermal melanocytes. They may develop on anatomical regions such as hands or feet, and, less commonly, on the head, neck, trunk and buttocks [1]. Their occurrence at extra-cutaneous sites including meninges, orbit, conjunctiva, maxillary sinus, oral mucosa, spermatic cord, prostate, lymph nodes, uterine cervix, and vagina are less reported [2].

The classification of blue nevi is complex, with biological behavior being benign, borderline, or malignant [3]. Only a few cases of giant cellular blue nevus (greater than 5 cm) have been reported [4,5]. The angiomatoid cellular blue nevus is a variant of blue nevus that is rarely reported in the literature [6]. Herein, we describe an unusual case of angiomatoid giant cellular blue nevus that arises from the vagina of a young woman during pregnancy, and present the ultrasonographic, gross, microscopic, and immunohistochemical findings.

### Clinical case

**Figure 1:**
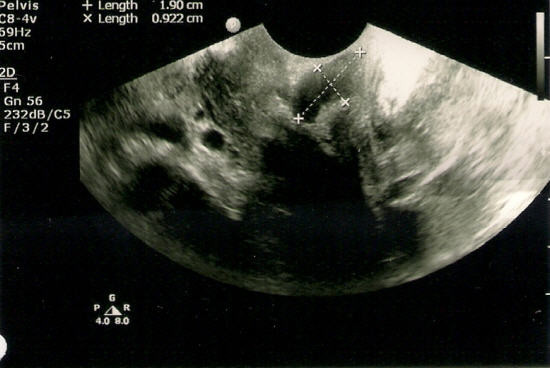
**Pelvic ultrasound at the 17^th ^week of pregnancy**. There was 1.9 × 0.9 cm oval hypoechoic bilobed area overlaying introitus.

**Figure 2:**
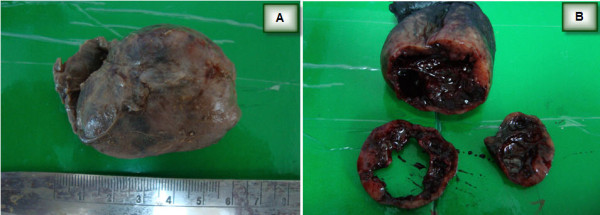
**Gross photograph**. **(A) **The tumor mass measuring 6.0 × 4.3 × 3.5 cm with the focal hyperpigmented external surface. **(B) **A cross section showing the central cystic change filled with dark friable material.

**Figure 3:**
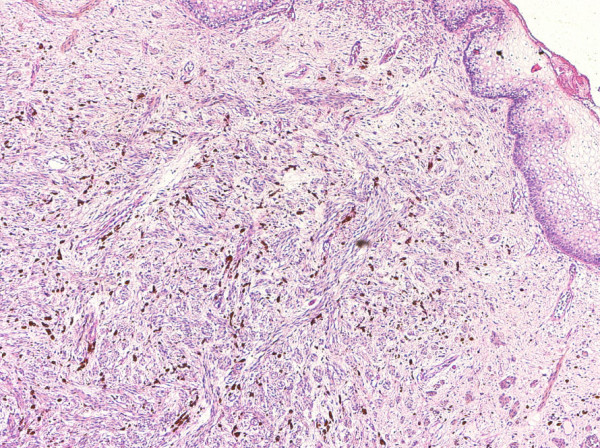
**Photomicrograph of the vaginal mass**. There is a subepithelial proliferation of spindle cells, accompanied by melanophages (original magnification, × 40, hematoxylin and eosin stain).

A 29-year-old woman was presented for routine antenatal care, and was found to have a small pigmented lesion in the vagina that increased gradually in size during pregnancy. Gynecologic examination showed a bluish soft lesion on the anterior vaginal wall. A pelvic ultrasound was carried out on the 17^th ^week of pregnancy, and showed a 1.9 × 0.9 cm oval hypoechoic bilobed area overlaying introitus (Figure 1). This mass had increased three-fold in size by 37^th ^week of pregnancy, forming a protrusion, which occupied most of the vaginal canal. The clinical impression at this stage was vaginal hemangioma; therefore the patient was selected clinically for cesarean section due to the risk of bleeding. Preoperative laboratory investigations revealed a hemoglobin level of 12.6 g/dL, a white cell count of 12.1 × 10^3^/μL, and platelet levels of 193 × 10^9^/L in the patient. The renal and liver functions were normal. However, the mass was resected during cesarean section, and the patient had a postoperative course without complication. Gross examination showed a cystic mass measuring 6.0 × 4.3 × 3.5 cm. The outer surface had focal areas of dark pigmentation (Figure 2). Serial cuts revealed central cystic changes filled with dark friable material. The entire specimen was submitted for histologic evaluation. Microscopic examination revealed a subepithelial cellular proliferation of heavily pigmented dendritic melanocytes that were arranged predominantly in fascicles, and epithelioid nests dissected through the vaginal wall. These were surrounded by collagen fibers, and a prominent vascular stroma associated with frequent melanophages (Figure 3). However, no cytologic pleomorphism, atypical mitosis, or necrosis was found.

**Figure 4:**
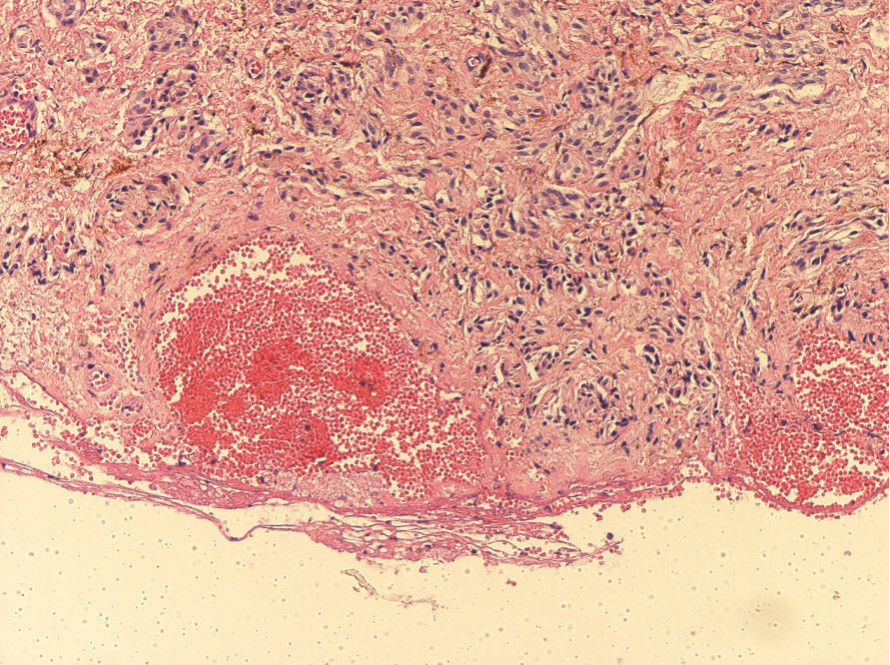
**Photomicrograph of the cavity**. There are a blood-filled, nonendothelial lined pseudovascular spaces surrounded by the dendritic melanocytes (original magnification, × 40, hematoxylin and eosin stain).

**Figure 5:**
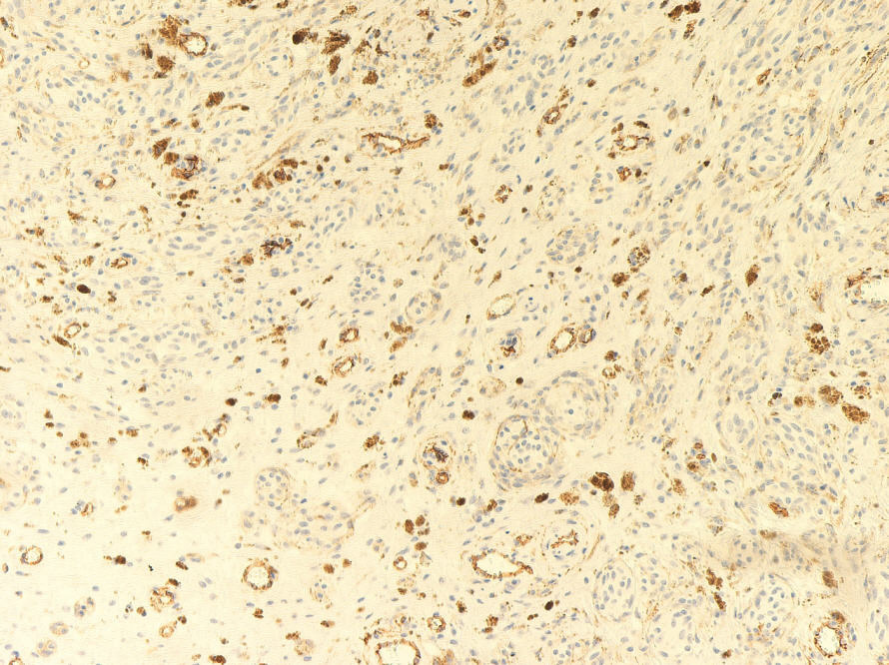
**CD31 immunohistochemical stain**. There are numerous small-sized vessels within the stroma of the cellular blue nevus.

There was no melanocytic junctional activity. The histology of the central cyst of the mass lesion revealed blood filled nonendothelial lined pseudovascular spaces that were lined by the lesional cells (in a manner analogous to an angiomatoid melanoma or aneurysmal benign fibrous histiocytoma) (Figure 4) Immunohistochemical analysis of the proliferation using the streptavidin-biotin immunohistochemical technique revealed that the tumor cells were positive for HMB-45, S-100 and melan-A, but negative for estrogen receptor, progesterone receptor, CD34, smooth muscle actin, and AE1/AE3. The MIB-1 proliferative index was less than 1%. CD31 and factor VIII immunohistochemical stains showed the presence of a prominent small-sized vascular network within the melanocytic proliferation (Figure 5). Clinical follow-up showed no evidence of recurrence at one year after the resection of the mass.

## Discussion

Blue nevi are distinct dendritic melanocytic proliferations that may arise in the skin [3] or Müllerian tract [2]. However, the origin of blue nevi in the Müllerian tract has been subject to controversy. Some authors [7] have proposed that these lesions may originate from melanocytic precursors that migrate towards the epithelium during embryogenesis. Other investigators have suggested that these lesions may result from the transformation of the stromal Schwann cells into melanocytes [2]. A possible source of these lesions in the vaginal wall is aberrantly migrated melanocytes. Nigogosyan et al. [8] recorded three cases in 100 autopsies that had dendritic cells located in the basal layer of the vaginal squamous epithelium. However, melanocytes were not recognized within the vaginal subepithelial stroma despite extensive sampling and utilization of the special stain, Fontana-Masson. This observation may indicate that a small proportion of women have ectopic melanocytes in the vagina, which may act as precursors for vaginal blue nevi and malignant melanomas.

Blue nevi have a wide spectrum of morphological variants. The common blue nevus and cellular blue nevus are the most frequent types [3]. Other morphologic types include (i) the atypical cellular blue nevus [9], (ii) the desmoplastic cellular blue nevus [10], (iii) the CD34-poistive cellular blue nevus [11], (iv) the epithelioid blue nevus [12], (v) the compound blue nevus [12], (vi) the amelanotic blue nevus [3], (vii) the congential pauci-melanotic cellular blue nevus [13], (viii) the sclerosing mucinous blue nevus [14], (ix) the cellular blue nevus with schwannian differentiation [3], and (x) the angiomatoid cellular blue nevus [6].

The angiomatoid variant is characterized by a vascular component and is described as an extremely rare variant [6].

The presence of a rich vascular component was described as a distinct variant of other tumors, including melanocytic and non-melanocytic lesions. The angiomatoid melanoma is a rare morphologic variant [15]. Angiomatoid dermatofibroma, angiolipoma, and glomangioma, are distinct morphologic variants for non-melanocytic tumors [16].

The occurrence of a cellular blue nevus in the vagina is extremely rare and, to the author's knowledge, only three cases have been published in the literature [2]. Giant cellular blue nevus (GCBN) has been described in extra-genital sites, such as the chest wall [5]. However, GCBN of the vagina has not been described previously.

Few studies have explored the influence of pregnancy on melanocytic lesions [17] or their size during pregnancy. The cause of enlargement of the melanocytic nevi during pregnancy is subject to controversy. Some authors [17] have proposed that these lesions may be related to the hormonal influence of gestation, although the immunohistochemical studies for the estrogen and progesterone receptors were negative. The increase in the size of the cellular blue nevus in the present case is more likely related to an increased vasculature within the mass lesion, as shown by the immunoreactivity of the prominent vascular network for CD31 and factor VIII immunohistochemical stains. This increase in the vascular component may provide a clinical impression of hemangioma. Furthermore, the presence of central cystic degeneration that was full of hemorrhagic material (Figure 2) may be another contributory factor to the increase in size of this mass during pregnancy.

## Conclusion

The angiomatoid giant cellular blue nevus of the vagina is extremely rare, and may clinically mimic hemangioma. The low proliferative index as shown by MIB-1 immunohistochemical marker and the absence of nuclear pleomorphism, or necrosis indicated benign histological features. This observation was supported by the absence of any recurrence at the clinical follow up after one year.

## Consent

Written informed consent was obtained from the patient for publication of this case report and any accompanying images. A copy of the written consent is available for review by the Editor-in-Chief of this journal.

## Abbreviations

GCBN: Giant cellular blue nevus

## Competing interests

The authors declare that they have no competing interests.

## Authors' contributions

The author prepared, read and approved the final manuscript
